# Dicing DICER-LIKE2 roles: Identification of siRNA-independent DICER-LIKE2 functions

**DOI:** 10.1093/plcell/koae078

**Published:** 2024-03-12

**Authors:** Nicolas M Doll

**Affiliations:** Assistant Features Editor, The Plant Cell, American Society of Plant Biologists; Laboratoire Reproduction et Développement des Plantes, University of Lyon, ENS de Lyon, UCB Lyon 1, CNRS, INRAE, F-69342, Lyon, France

Small interfering RNAs (siRNAs) play key roles in eukaryotic cells, such as repressing transposons and viruses, as well as controlling gene expression. siRNA biogenesis involves DICER-LIKE (DCL) proteins, a family of type III RNAses, composed of 4 members in Arabidopsis (*Arabidopsis thaliana*). Each DCL produces a specific kind of small RNA by processing double-strand RNAs (dsRNAs) (Margis et al. 2006). DCL2 produces 22 nucleotide-long siRNAs, unlike other DCLs that produce 21-nt (DCL1 and DCL4) or 24-nt (DCL3) small RNAs.

In plants, DCL2 has several described effects. It is important for systemic RNA interference and produces siRNA against specific RNA viruses (Fig. 1A), and together with DCL4, DCL2 activity confers basal antiviral resistance against non-adapted viruses. However, in the absence of DCL4, DCL2 induces non-fully penetrant growth reduction and leaf yellowing, which are suppressed in *dcl2 dcl4* double mutants (Fig. 1B) (Nielsen et al. 2024). Until now, it has been widely assumed that all DCL2 effects are related to its role in 22-nt siRNA-mediated silencing. Developmental defects occurring in *dcl4* mutants were postulated to originate from disturbances in 22-nt siRNA production because the dsRNA substrates typically processed by DCL4 would be available for DCL2. For example, some developmental defects observed in *dcl4* were proposed to result from ectopic inhibition by 22-nt siRNA of genes encoding nitrate reductases NIA1 and NIA2, as well as phloem differentiation factors SMXL4 and SMXL5 (Zhang et al. 2015; Wu et al. 2017). However, despite being commonly assumed, the hypothesis that 22-nt siRNA silencing causes the DCL2 effects has never been properly confirmed. In new work, **Carsten Poul Skou Nielsen, Laura Arribas-Hernández, and colleagues** (Nielsen et al. 2024) investigated the link between 22-nt siRNA produced by DCL2 and the roles of DCL2 in basal antiviral resistance and development in Arabidopsis.

**Figure 1.**
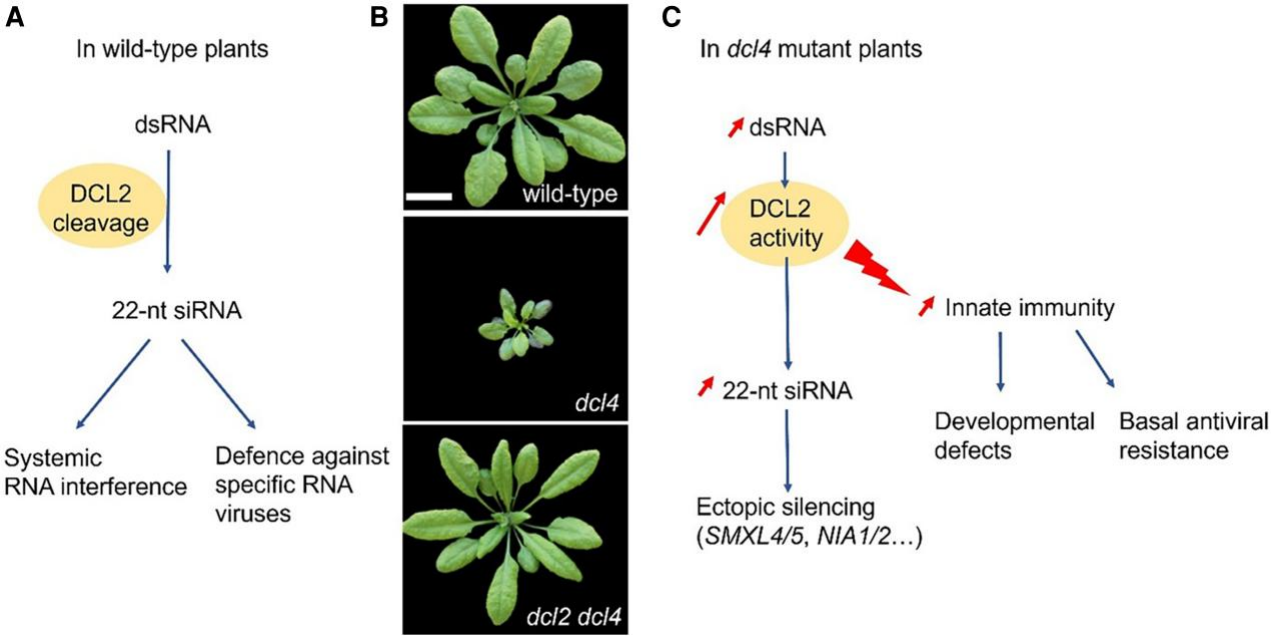
DCL2 effects in wild-type and *dcl4* mutant plants. **A)** DCL2 functions in wild-type plants. **B)** Twenty-six-day-old wild-type, *dcl4*, and *dcl2 dcl4* mutant Arabidopsis plants. Scale bar = 2 cm. **C)** DCL4 absence leads to new DCL2 functions. B was adapted from Nielsen et al. (2024), Figure 2.

First, the authors analyzed the occurrence of the developmental defects caused by DCL2 activity in some *dcl4* mutants. The appearance of growth defects and chlorosis required the complete absence of DCL4 activity, accessibility to dsRNA, and full RNAse III and helicase activities of DCL2, with the latter also needed for basal antiviral response. Unexpectedly, they also showed that a mere 2-fold decrease in DCL2 protein content, observed in *dcl2* heterozygous plants, suppressed these developmental defects and was sufficient to cause increased susceptibility to a disarmed virus in the absence of DCL4. Interestingly, DCL2 heterozygosity did not substantially decrease 22-nt siRNA production, including the production of siRNA targeting *NIA1/2* and *SMX4/5*, thus indicating that unlike the aforementioned effects of DCL2, normal siRNA production does not require full DCL2 dosage. This observation indicates that basal antiviral resistance and developmental defects are, at least partially, caused by something other than the 22-nt siRNAs produced by DCL2.

To further investigate the link between DCL2 dosage and developmental defects, DCL2 was expressed at different levels in *dcl2 dcl4* mutants. Remarkably, the odds of plants displaying developmental defects correlate with DCL2 protein level over a 20-fold expression range, suggesting that it is the catalytic activity of DCL2 rather than the silencing activity of the siRNAs produced that may cause the developmental alterations.

Finally, the authors analyzed 4 point-mutations in the DCL2 helicase domain. Upon loss of DCL4, these mutants display reduced basal antiviral resistance and the absence of developmental defects but still produced 22-nt siRNAs, again uncoupling the production of 22-nt siRNAs in general from these DCL2 effects. However, the big picture is more complicated as many of the 22-nt siRNAs produced in the absence of DCL4 differed from those produced by DCL2 in wild-type plants.

In summary, the effects of DCL2 in the absence of DCL4, namely conferring basal antiviral resistance and causing developmental defects, can be uncoupled, at least partially, from the 22-nt siRNAs produced by DCL2. What, then, could cause these effects? The authors proposed that in the absence of DCL4, the accumulation of dsRNA (normally processed by DCL4) increases DCL2 catalytic turnover. DCL2 catalytic turnover triggers an innate immunity response, conferring basal antiviral resistance to the plant and causing the developmental defects observed (Fig. 1C). Strong support for this hypothesis comes from an associated paper showing that mutations in 2 intracellular immune receptors of the nucleotide binding leucine-rich repeat class suppress the DCL2-mediated developmental defects (Nielsen et al. 2023). What are the sensors perceiving DCL2 catalytic turnover and how do they activate innate immunity are new questions raised by this study.

## Dive Curated Terms

The following phenotypic, genotypic, and functional terms are of significance to the work described in this paper:

dcl4 Gramene: AT5G20320
dcl4 Araport: AT5G20320

DCL3 Gramene: AT3G43920
DCL3 Araport: AT3G43920

NIA1 Gramene: AT1G77760
NIA1 Araport: AT1G77760

NIA2 Gramene: AT1G37130
NIA2 Araport: AT1G37130

DCL2 Gramene: AT3G03300
DCL2 Araport: AT3G03300

DCL1 Gramene: AT1G01040
DCL1 Araport: AT1G01040
